# Horner’s Syndrome and Upper Limb Paresthesia During Labor Epidural Analgesia: A Case Report

**DOI:** 10.7759/cureus.21388

**Published:** 2022-01-18

**Authors:** João Crisóstomo, Carolina Dias, Daniel Pedro, Rafael Pires, Teresa Rocha

**Affiliations:** 1 Anesthesiology, Centro Hospitalar Universitário Lisboa Central, Lisbon, PRT

**Keywords:** analgesia, labour epidural analgesia, epidural, horner’s syndrome after epidural, horner’s syndrome

## Abstract

Horner’s syndrome is a condition that results from sympathetic nervous system dysfunction. Labor epidural analgesia is known to be a rare cause of Horner’s Syndrome. However, in the obstetric population, the incidence of Horner’s Syndrome is higher than in the rest of the population as it is a consequence of high cephalad spread of local anesthetic (LA) probably enhanced by the anatmophysiologic changes of pregnancy. We present a case of unilateral Horner’s syndrome as a complication of epidural analgesia with accompanying upper limb paresthesia and motor weakness, a rarely encountered symptom.

## Introduction

Horner’s syndrome, also known as oculosympathetic palsy, is a condition that results from sympathetic nervous system dysfunction. It consists of a classic triad of ptosis, miosis, and anydrosis of the affected side and is usually a benign, self-limited condition once the local anesthetic (LA) is metabolized [1]. Labor epidural analgesia is known to be a rare cause of Horner’s syndrome but in the obstetric population, the incidence is higher than in the rest of the population [1]. There are several anatomical and physiological changes during pregnancy that facilitate high cephalad spread of LA such as the narrowing of the epidural space due to venous engorgement and the further transient increase in epidural pressure because of uterine contractions. Furthermore, increasing progesterone levels sensitize neuronal tissue facilitating the blockage of the sympathetic nervous system [1]. Among other causes of Horner’s syndrome are head and neck surgery, tumors or trauma, and several conditions affecting the internal carotid artery or the internal jugular vein [2].

The aim of this case report was to present a case of recurrent Horner’s syndrome with accompanying upper limb paresthesia and motor weakness, a rarely encountered symptom, after labor epidural analgesia.

## Case presentation

**Figure 1:**
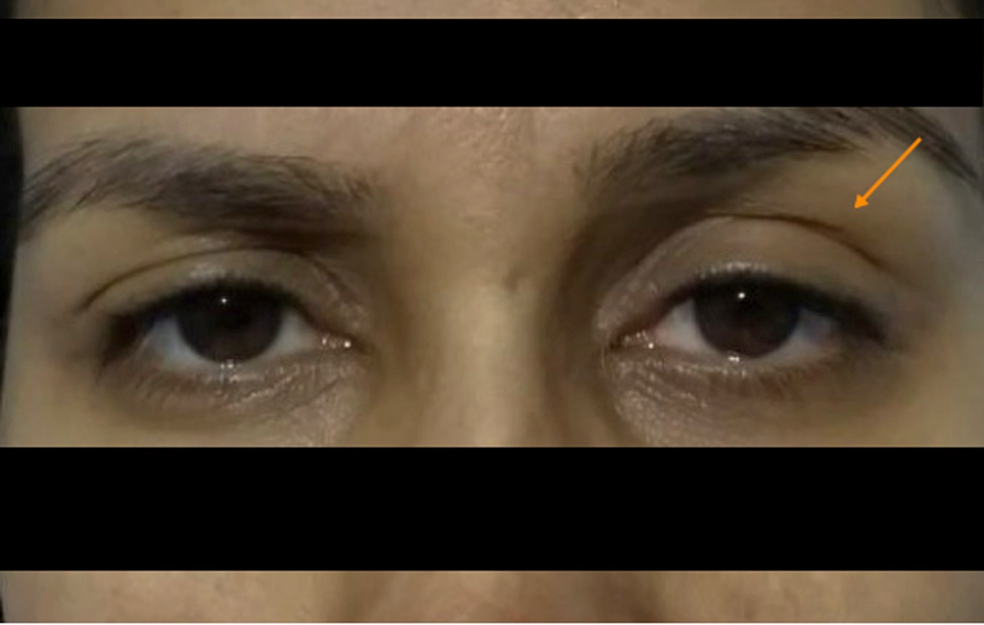
Ptosis of left eyelid and miosis of the ipsilateral eye.

A 35-year-old woman with a body mass index (BMI) of 24.2, 165cm in height, and 66 kg weight, primigravida with a gestational age of 38 weeks and three days presented to the obstetric emergency department because of premature rupture of membranes. She had a medical history of iron deficiency anemia compensated with iron supplementation. She did not have any other comorbidities or allergies. Initially, labor was induced with oral misoprostol and later with perfusion of oxytocin. The patient requested neuroaxial analgesia approximately nine hours after entering the labor room. She was in active labor with a visual analog scale (VAS) score of 7/10. A combined spinal-epidural analgesia technique was performed. A 18G Tuohy needle was used to find the epidural space at the L3-L4 interspace in the sitting position using a loss of resistance to saline technique. A 27G pencil-point spinal needle was introduced through the Tuohy needle into the subarachnoid space. Cerebrospinal fluid return confirmed correct positioning and a dose of Hypobaric levobupivacaine 0.25%, 0.5 mL (1.25 mg) + sufentanil 5 µg was administered intrathecally. A 18G epidural catheter was then advanced and fixed at a depth of 9 cm in the skin keeping 4.5 cm in the epidural space. She maintained a supine position with a slight head and torso elevation for the following 10 minutes. After five minutes VAS score was 3/10. No motor block or other neurological symptoms were reported. Pain relief lasted two hours, after which the patient requested further analgesia. After negative aspiration of the catheter to rule out blood or cerebrospinal fluid return, a 3-mL test dose of 0.2% ropivacaine was given and intratecal injection was excluded, as there was no motor loss. Then, 10 mL ropivacaine 0.2% and 10 µg sufentanil were injected. Twenty minutes after injection the patient complained of paresthesia around the left eye and in the left hand. Clinical examination revealed unilateral miosis of the left eye, with left upper eyelid ptosis (Figure 1) and an ipsilateral decrease in handgrip strength. The sensory block level was at the intermammillary region (T4). No other neurological findings were detected. The Parturient´s arterial pressure was 120-60 mmHg, and the cardiotocography showed good fetal cardiac frequency and variability. Symptoms gradually faded over the next two hours. As the pain returned another epidural bolus was given using a reduced volume (8 mL) and concentration of LA (ropivacaine 0,15%) without any opioid. Again, approximately 20 minutes after, the symptoms recurred. Similarly, no clinically significant hypotension was associated with the block. No further boluses were given until delivery and resolution of symptoms occurred three hours after the last bolus.

The patient denied any symptoms in the following days, including headache, and was discharged home three days after delivery.

## Discussion

The Horner’s phenomenon results from blockage of sympathetic preganglionic fibers of the face and eye originating from the anterior horn cells of C8 and T1, and occasionally as low as T4.

We hypothesize that a rupture in the dura mater during the subarachnoid puncture of the initial combined spinal-epidural technique may have created a larger than usual communication with the epidural space, allowing for greater intrathecal spread during both epidural boluses. On the other hand, an atraumatic pencil-point needle was used, which should theoretically reduce the damage to the dura mater fibers [3]. Although not mandatory, a postdural puncture headache could have been present, associated with a larger tear on the dura mater, which was not the case. The anatomical and physiological changes of pregnancy must have also contributed to the higher spread of LA which presumably reached the C7-T1 level, resulting in paresthesia and some degree of a motor block of the brachial plexus. This mechanism of increased epidural spread of LA due to higher epidural pressures has also been reported in pediatric patients with scoliosis and abnormal narrowing along the epidural space, following epidural anesthesia [4].

In a recent review of the literature which included 56 cases of Horner’s syndrome, 18 had altered ipsilateral arm sensory or motor function [2]. The ipsilateral paresthesia of the upper arm is difficult to explain, and the presence of a midline septum has been suggested as the cause [5]. A midline septum could have also contributed to the narrowing of the epidural space facilitating LA ascension. Another possible explanation could be related to the baricity of the LA and being a phenomenon gravity-dependent. The LA used is isobaric at room temperature, becoming hypobaric at body temperature. Hypobaric sufentanil further decreases the baricity of the mixture [6]. Both boluses were administered with the patient in the sitting position, which is not consistent with a unilateral block.

The symptoms are typically transient with an average onset of 25 minutes, and a mean duration of 215 minutes (range: several minutes to 24 hours) until spontaneous resolution [7]. In our case symptoms appear approximately 20 minutes after each bolus and disappear three hours after the administration of LA, which is concordant with the literature.

## Conclusions

Prompt diagnosis of Horner’s syndrome or oculosympathetic palsy should alert the anesthesiologist for the possibility of a cephalic spread of LA. Atypical symptoms such as upper limb paresthesia may indicate an even higher blockage. The diagnosis is also important in order to reassure the patient of its transient and benign condition. Nevertheless, anesthesiologists should be aware that cardiopulmonary arrest may occur as a result of total spinal anesthesia. Persistent Horner’s syndrome should warrant further evaluation to rule out other diagnoses.
